# Formulation of Hyperelastic Constitutive Model for Human Periodontal Ligament Based on Fiber Volume Fraction

**DOI:** 10.3390/ma18030705

**Published:** 2025-02-06

**Authors:** Bin Wu, Chenfeng Huang, Na Li, Yi Lu, Yang Yi, Bin Yan, Di Jiang

**Affiliations:** 1 College of Mechanical and Electronic Engineering, Nanjing Forestry University, Nanjing 210037, China; wubin@njfu.edu.cn (B.W.); hcf980615@163.com (C.H.); juliusx@163.com (Y.L.); yiyang_njfu@163.com (Y.Y.); 2 Department of Orthodontics, Affiliated Hospital of Stomatology, Nanjing Medical University, Nanjing 210029, China; 3 Jiangsu Province Key Laboratory of Oral Diseases, Nanjing 210029, China; 4 Jiangsu Province Engineering Research Center of Stomatological Translational Medicine, Nanjing 210029, China

**Keywords:** human periodontal ligament, collagen fiber, volume fraction, hyperelastic model, uniaxial tensile

## Abstract

Collagen fibers of the Periodontal ligament (PDL) play a crucial role in determining its mechanical properties. Based on this premise, we investigated the effect of the volume fraction of human PDL collagen fibers on the hyperelastic mechanical behavior under transient loading. Samples were obtained from different root regions (neck, middle, and apex) of the PDL, prepared from fresh human anterior teeth. The collagen fibers volume fraction in various regions of the PDL was quantified by staining techniques combined with image processing software. The collagen fiber volume fractions were found to be 60.3% in the neck region, 63.1% in the middle region, and 52.0% in the apex region. A new hyperelastic constitutive model was constructed based on the volume fraction. A uniaxial tensile test was conducted on these samples, and the accuracy of the constitutive model was validated by fitting the test data. Also, relevant model parameters were derived. The results demonstrated that human PDL exhibited hyperelastic mechanical properties on the condition of transient loading. With an increase in the volume fraction of collagen fibers, the tensile resistance of the PDL was enhanced, demonstrating more significant hyperelastic mechanical properties. The hyperelastic constitutive model showed a good fit with the experimental results (R^2^ > 0.997), describing the hyperelastic mechanical properties of the human PDL effectively.

## 1. Introduction

The Periodontal ligament (PDL) is a dense connective tissue wrapped around the root of the tooth [1,2,3]. In orthodontic treatment, the PDL transmits orthodontic forces to the alveolar bone, facilitating tooth movement [4,5,6,7]. Therefore, the mechanical response of the PDL plays a key role in orthodontic treatment [8,9]. The PDL tissue is composed of biological tissues such as collagen fiber, blood vessels, and matrix, among which the collagen fiber is the primary component [10,11,12]. The ability of the PDL to resist transient loading is determined by collagen fibers [13,14]. Therefore, quantifying and exploring the specific effect of collagen fibers on the human PDL resistance to transient load is the key to evaluating the influence of orthodontic force accurately on the mechanical response of the PDL. It is of great significance for understanding the mechanical mechanisms of orthodontic treatment.

Both human and animal PDL have distinct hyperelastic mechanical properties and have been studied by many researchers. Zhou et al. [15] conducted mechanical tests on the pig PDL. Finding that the third-order Ogden hyperelastic model could describe its hyperelastic mechanical properties effectively. Nikolaus et al. [16] used the hyperelastic model to analyze the effect of human PDL thickness and geometry on masticatory function, verifying the accuracy of this method through tests. Wu J et al. [17] proposed a new constitutive model to describe the hyperelastic mechanical properties of the human PDL. Karimi et al. [18] gave the PDL hyperelastic material properties and found that the stress of the PDL was concentrated in the cervical margin.

At present, most studies on PDL have not considered the influence of collagen fiber volume fraction on its hyperelastic mechanical properties. Liu et al. [19] used optical microscopy, three-dimensional reconstruction, Raman spectroscopy, and other methods to reveal the composition and biomechanical properties of human PDL collagen fibers. It was found that the collagen fiber volume fraction had a great influence on the tensile strength of PDL. Connizzo, BK et al. [20] found that the special function of PDL is caused by the non-uniform structure of the tissue. Hirashima, S et al. [21] revealed the three-dimensional ultrastructure of the PDL collagen bundle by scanning electron microscope tomography, showing that the PDL bundle is a multi-branch structure wrapped in PDL fibroblasts cytoplasmic sheet. We have performed nanoindentation tests on the human PDL samples and established the relevant viscoelastic constitutive model in our previous research [22]. The volume fraction of collagen fibers had a great influence on the viscoelastic mechanical properties of the PDL. The larger the volume fraction, the more obvious its viscoelastic mechanical properties. However, this research only explained its viscoelastic mechanical properties, ignoring the hyperelastic mechanical properties of the PDL under transient load.

To reveal the influence of collagen fiber volume fraction on the hyperelastic mechanical properties of the PDL, this research measured the volume fraction of collagen fibers in different regions of the PDL through microscopic observation. Combined with the uniaxial tensile test, a hyperelastic constitutive model of the PDL based on the collagen fiber volume fraction was constructed. These findings contribute to a deeper understanding of the microscopic mechanical properties of human PDL, while also providing guidance for predicting and analyzing tooth movement during clinical orthodontic treatment.

## 2. Materials and Methods

### 2.1. Sample Preparation

**Figure 1:**
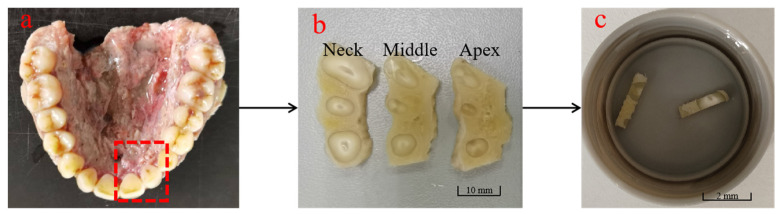
Sample preparation of PDL. (**a**) Select the biological tissues from the central and lateral incisors; (**b**) transverse section perpendicular to the long axis of the tooth; (**c**) experimental samples.

The samples were obtained from the jawbone of a fresh human cadaver (male, 60 years old). A saw (12-inch, SATA, Nanjing, China) was used to segment the tissue of the central and lateral incisors used in the test. The crown was removed using a low-speed cutter (500 r/min, Isomet, Bueler, Lake Bluff, IL, USA), and the remaining parts were cut in the direction perpendicular to the tooth-long axis to obtain samples perpendicular to the tooth-long axis. These samples were divided into the neck, middle, and apex of the tooth [23], and then further dissected to obtain rectangular specimens suitable for uniaxial tensile tests. The sample preparation process is shown in Figure 1. This research has been reviewed and approved by the Institutional Review Board (IRB) of Nanjing Medical University (No. (2020) 234).

Each sample is stored in separate containers after cutting to differentiate samples from various regions, with the orientation of each section clearly labeled. All samples were immersed in normal saline and stored at −20 °C in a dark environment to preserve the integrity of the biological structure and the stability of the samples’ biological properties. The dimensions of PDL samples are listed in Table 1.

### 2.2. PDL Section Staining and Collagen Fiber Volume Fraction Measurement

Masson staining is one of the primary methods for collagen fiber staining in tissues. After Masson staining, the collagen fibers in the PDL appear blue, while the rest of the tissue appears red. The middle incisor and lateral incisor from the opposite side of the same jawbone were selected to prepare samples for Masson staining. After fixation, tissue decalcification, dehydration, embedding, and other treatments, the tooth was sliced along the long axis of the tooth. The section thickness was 4 μm. Five suitable and undamaged sections were selected for Masson staining.

**Figure 2:**
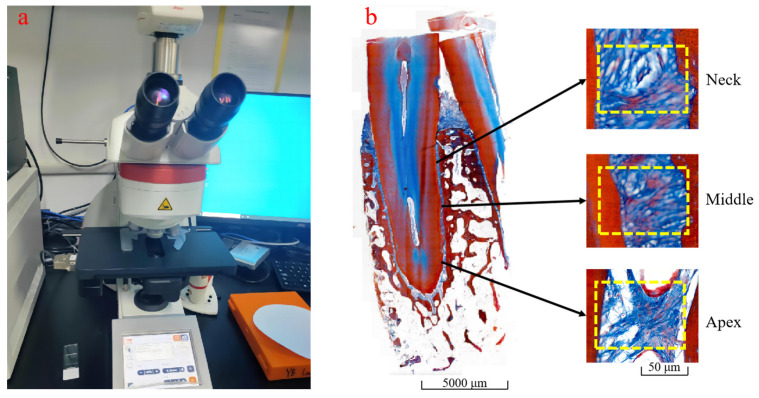
Collagen fiber volume fraction measurement. (**a**) Color digital microcamera; (**b**) ROI selecting from different regions.

Color digital microcamera (DMC5400 series, Leica Microsystemsl, Heerbrugg, Switzerland) was used to photograph the tooth sections after Masson staining, facilitating the subsequent quantitative analysis and measurement of the volume fraction of human PDL collagen fibers. Region of Interest (ROI) was selected by Image processing software Image J (V1.8, National Institutes of Health, Bethesda, MD, USA) for measurement and calculation in different regions of the human PDL, namely the apex, middle, and neck, as shown in Figure 2. $P_{x}$ represents collagen fiber area, and $P_{I}$ represents ROI area. Human PDL collagen fiber volume fraction ($V_{f}$) was calculated as follows:

(1) $$V_{f}=P_{x}\cdot {\left(P_{I}\right)}^{-1}$$

### 2.3. Uniaxial Tensile Test

**Figure 3:**
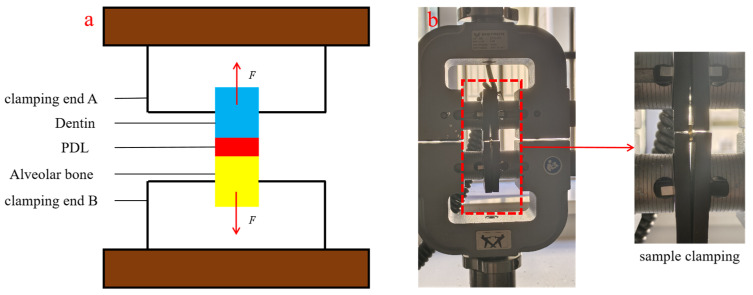
Uniaxial tensile test. (**a**) Schematic diagram of the uniaxial tensile test; (**b**) actual setup of the uniaxial tensile test.

Experiments were conducted using a high-precision dual-column electronic universal testing machine (Instron 3365 series, Instron, Boston, MA, USA). The tensile rates were set at 0.01 mm/min, 0.05 mm/min, and 0.1 mm/min. It was essential to avoid inconsistencies in the distance between the clamping ends A and B on both sides of the human PDL tissue sample during the clamping process. The preload tests were performed on the human PDL samples before the formal experiments to mitigate the influences of tissue relaxation after prolonged static holding. The pre-loading frequency was set at 1 Hz, which corresponds to the average human chewing frequency [24]. The pre-loading strain was set at 0.1 given that the strain damage threshold of the human PDL is approximately 0.35 [25,26], and the pre-loading tests were repeated for 20 cycles. Uniaxial tensile tests were conducted following the completion of the preload tests. Figure 3 illustrates both the schematic and actual setup of the uniaxial tensile test.

## 3. Constitutive Model Based on the Volume Fraction of Collagen Fibers

Observations of the PDL samples reveal that the orientation of collagen fibers aligns with the direction of applied force after preload tests. So, it is appropriate to choose a hyperelastic constitutive model with transverse isotropy. The hyperelastic constitutive model of PDL can be expressed by establishing a relationship between the Cauchy–Green deformation tensor (C) and the invariants of the strain tensor ($I_{i}$) on the basis of the strain energy function.

(2) $$W(C)=W(I_{1},I_{2},I_{3})+W(I_{4},I_{5})$$

Formula (2) shows that $V_{m}$ represents the volumetric fraction of the remaining biological tissues in the PDL, while $V_{f}$ denotes the volume fraction of the collagen fibers in the PDL, $V_{m}+V_{f}=1$. $W_{m}$ and $W_{f}$ correspond to the mechanical characteristics of the remaining biological tissues and the collagen fibers, respectively. The relationship for the invariants of the strain tensor ($I_{i}$) is expressed as follows:

(3) $$\left\{\begin{array}{l} I_{1}=\mathrm{tr}(C)\\ I_{2}=1/2\left[{\left(\mathrm{tr}(C)\right)}^{2}-\mathrm{tr}(C{)}^{2}\right]\\ I_{3}=\det(C)\\ I_{4}=N\cdot C\cdot N^{T}=\lambda_{F}^{2}\\ I_{5}=N\cdot C^{2}\cdot N^{T} \end{array}\right.$$

Formula (3) shows that N describes the directional characteristics of collagen fibers in the absence of applied load, while $\lambda_{F}$ represents the elongation rate of the collagen fibers, reflecting the extent of deformation when the fibers are under stress. $I_{1}$ describes the deformation behavior of the PDL under tensile conditions. $I_{3}$ is related to the volumetric changes in the material. Based on the assumption of incompressibility of the PDL, it can be defined that $I_{3}=1$. $I_{4}$ is associated with the tensile strain energy of the fibers and increases with the increase of $\lambda_{F}$. For PDL, the influence of the matrix on the fiber during uniaxial tensile deformation can be ignored, and the interaction between matrix and fiber is small, so $I_{2}$ and $I_{5}$ can be approximately ignored [27].

The PDL samples were subjected to stress only in the X direction, while no stress is applied in the Y,Z directions in the uniaxial tensile test. The elongation rates in the three principal directions, X,Y,Z are denoted as $\lambda_{1},\lambda_{2},\lambda_{3}$, respectively. Therefore, the deformation gradient tensor (F) of the PDL can be expressed as follows:

(4) $$F={\left(\frac{\partial x_{i}}{\partial a_{j}}\right)}_{i,j=1,2,3}=\left[\begin{array}{ccc} \lambda_{1} & 0 & 0\\ 0 & \lambda_{2} & 0\\ 0 & 0 & \lambda_{3} \end{array}\right]$$

$\left(a_{1},a_{2},a_{3}\right)$ represents the coordinates of a point in the PDL before deformation due to stress, while $\left(x_{1},x_{2},x_{3}\right)$ represents the coordinates of the same point after deformation in the Formula (3). Consequently, the Cauchy–Green deformation tensor (C) and the first tensor invariant ($I_{1}$) can be expressed as follows:

(5) $$C=F^{T}F=\left[\begin{array}{ccc} \lambda_{1}^{2} & 0 & 0\\ 0 & \lambda_{2}^{2} & 0\\ 0 & 0 & \lambda_{3}^{2} \end{array}\right]$$

(6) $$I_{1}=\mathrm{tr}(C)=\lambda_{1}^{2}+\lambda_{2}^{2}+\lambda_{3}^{2}$$

The strain energy function related to the first tensor invariant ($I_{1}$) and the fourth tensor invariant ($I_{4}$) can be expressed as follows:

(7) $$W=V_{m}c_{1}\left(e^{Q_{1}}-1\right)+V_{f}c_{2}\left(e^{Q_{2}}-1\right)$$

(8) $$Q_{1}=c_{3}{\left(I_{1}-3\right)}^{2}+c_{4}{\left(\alpha -1\right)}^{4}$$

(9) $$Q_{2}=c_{5}{\left(I_{1}-3\right)}^{2}+c_{6}{\left(\alpha -1\right)}^{4}$$

(10) $$I_{4}=\alpha^{2}$$

Formula (10) represents the particle coordinates of the PDL when it is not subjected to external forces. $I_{4}=N\cdot C\cdot N^{T}$, N is the unit vector. $N=\left[\begin{array}{ccc} 1 & 0 & 0 \end{array}\right]$. Then, the formulas can be deformed into

(11) $$I_{4}=N\cdot C\cdot N^{T}=\left[\begin{array}{ccc} 1 & 0 & 0 \end{array}\right]\left[\begin{array}{ccc} \lambda_{1}^{2} & 0 & 0\\ 0 & \lambda_{2}^{2} & 0\\ 0 & 0 & \lambda_{3}^{2} \end{array}\right]\left[\begin{array}{c} 1\\ 0\\ 0 \end{array}\right]=\lambda_{1}^{2}$$

(12) $$\alpha =\sqrt{I_{4}}=\sqrt{\lambda_{1}^{2}}=\lambda_{1}$$

Formula (2) can be deformed by substituting the general strain energy function expression [23,28]:

(13) $$W=V_{m}c_{1}\left[e^{c_{3}{\left(I_{1}-3\right)}^{2}+c_{4}\left({\lambda_{1}-1}^{4}\right)}-1\right]+V_{f}c_{2}\left[e^{c_{5}{\left(I_{1}-3\right)}^{2}+c_{6}\left({\lambda_{1}-4}^{4}\right)}-1\right]$$

In the uniaxial tensile tests, the relationship between $\lambda_{1},\lambda_{2},\lambda_{3}$ can be expressed as

(14) $$\lambda_{3}^{2}=\lambda_{2}^{2}=\frac{1}{\lambda_{1}}$$

Based on the relationship between strain ratio and strain, it follows that $\lambda ={\left(1+2\varepsilon \right)}^{1/2}$ [29]. It can be defined that $\lambda_{1}=\lambda$. Thus, the first tensor invariant ($I_{1}$) and the fourth tensor invariant ($I_{4}$) to be expressed as

(15) $$I_{1}=\lambda^{2}+\frac{2}{\lambda }=\left(1+2\varepsilon \right)+\frac{2}{{\left(1+2\varepsilon \right)}^{1/2}}$$

(16) $$I_{4}=1+2\varepsilon$$

Taking the derivative of the strain energy potential (W) with respect to the right Cauchy–Green strain tensor (C) yields the second Piola–Kirchhoff stress tensor (S). Due to the incompressibility of the PDL, a pressure term (p) should be subtracted to enforce this condition.

(17) $$S=2\frac{\partial W}{\partial C}-pC^{-1}=2\left(\frac{\partial W}{\partial I_{1}}\frac{\partial I_{1}}{\partial C}+\frac{\partial W}{\partial I_{4}}\frac{\partial I_{4}}{\partial C}\right)-pC^{-1}$$

The partial derivative of strain energy (W) respect to the tensor invariants is

(18) $$\left\{\begin{array}{l} \frac{\partial W}{\partial I_{1}}=V_{m}c_{1}e^{Q_{1}}\cdot 2c_{3}\left(I_{1}-3\right)+V_{f}c_{2}e^{Q_{2}}\cdot 2c_{5}\left(I_{1}-3\right)\\ \frac{\partial W}{\partial I_{4}}=2V_{m}c_{1}e^{Q_{1}}\left[I_{4}^{-1/2}\cdot c_{4}{\left(I_{4}^{1/2}-1\right)}^{3}\right]+2V_{f}c_{2}e^{Q_{2}}\left[I_{4}^{-1/2}\cdot c_{6}{\left(I_{4}^{1/2}-1\right)}^{3}\right] \end{array}\right.$$

The partial derivative of the tensor invariants with respect to the Cauchy–Green strain tensor is

(19) $$\left\{\begin{array}{l} \frac{\partial I_{1}}{\partial C}=E\\ \frac{\partial I_{4}}{\partial C}=N⊗N \end{array}\right.$$

E represents the second-order identity tensor. The Cauchy stress (σ) that characterizes the actual stress is given by

(20) $$\sigma =J^{-1}\cdot F\cdot S\cdot F^{T}$$

J is the volume ratio of the material after deformation to that before deformation. Due to the incompressibility of the periodontal ligament material, J=1. Therefore, it can be concluded that

(21) $$\sigma =F\cdot S\cdot F^{T}$$

In the uniaxial tensile test, there is stress only in the principal direction X, and there is no stress in the other direction. Therefore, it can be concluded that:

(22) $$\left\{\begin{array}{l} \sigma_{11}\neq 0\\ \sigma_{22}=\sigma_{33}=0 \end{array}\right.$$

$\sigma_{11}$, $\sigma_{22}$ and $\sigma_{33}$ represent the true stress in the X,Y and Z direction. Under uniaxial tensile conditions, the stress perpendicular to the loading direction is 0. By combining Formula (17)–(22), the pressure term (p) can be derived that

(23) $$p=\frac{1}{\lambda }\left[\begin{array}{l} 4V_{m}c_{1}e^{c_{3}{\left(\lambda^{2}+\frac{2}{\lambda }-3\right)}^{2}+c_{4}{\left(\lambda -1\right)}^{4}}\cdot c_{3}\left(\lambda^{2}+\frac{2}{\lambda }-3\right)\\ +4V_{f}c_{2}e^{c_{5}{\left(\lambda^{2}+\frac{2}{\lambda }-3\right)}^{2}+c_{6}{\left(\lambda -1\right)}^{4}}\cdot c_{5}\left(\lambda^{2}+\frac{2}{\lambda }-3\right) \end{array}\right]$$

By combining Formulas (13)–(23), $\sigma_{11}$ can be derived that

(24) $$\begin{array}{ll} \sigma_{11} & =\left(\lambda^{2}-\frac{1}{\lambda }\right)\left[\begin{array}{l} 4V_{m}c_{1}e^{c_{3}{\left(\lambda^{2}+\frac{2}{\lambda }-3\right)}^{2}+c_{4}{\left(\lambda -1\right)}^{4}}\cdot c_{3}\left(\lambda^{2}+\frac{2}{\lambda }-3\right)\\ +4V_{f}c_{2}e^{c_{5}{\left(\lambda^{2}+\frac{2}{\lambda }-3\right)}^{2}+c_{6}{\left(\lambda -1\right)}^{4}}\cdot c_{5}\left(\lambda^{2}+\frac{2}{\lambda }-3\right) \end{array}\right]\\ & +4c_{4}\lambda {\left(\lambda -1\right)}^{3}\cdot V_{m}c_{1}e^{c_{3}{\left(\lambda^{2}+\frac{2}{\lambda }-3\right)}^{2}+c_{4}{\left(\lambda -1\right)}^{4}}\\ & +4c_{6}\lambda {\left(\lambda -1\right)}^{3}\cdot V_{f}c_{2}e^{c_{5}{\left(\lambda^{2}+\frac{2}{\lambda }-3\right)}^{2}+c_{6}{\left(\lambda -1\right)}^{4}} \end{array}$$

## 4. Results

### 4.1. Observation Results of Collagen Fiber Volume Fraction

#### 4.1.1. Microscopic Observation of Periodontal Collagen Fibers

**Figure 4:**
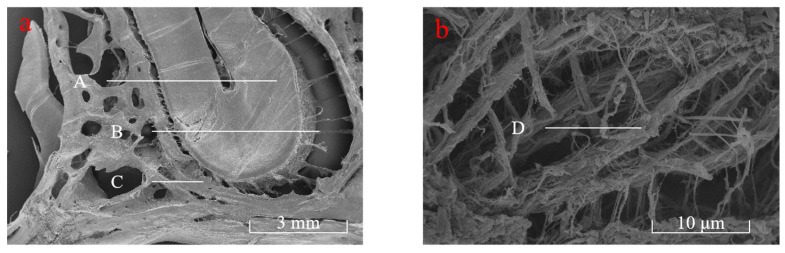
Electron microscopy observation diagram of PDL. (**a**) A: dentin, B: collagen fiber, C: alveolar bone; (**b**) D: fiber bundle.

The PDL samples were observed by electron microscopy, and the results are shown in Figure 4. This Figure reveals that the collagen fibers in the PDL combine the teeth with the alveolar bone as a whole organically. Collagen fibers consist of multiple bundles rather than single fibers. After preloading, these bundles aligned along the direction of force, demonstrating consistent alignment across different groups. This also verified that the collagen fibers in the PDL provided the primary resistance to loading under transient loading conditions [2,13].

#### 4.1.2. Imaging Analysis Results of Collagen Fiber Volume Fraction of PDL

Through imaging measurements, the volume fraction of collagen fibers in different regions of the human PDL is shown in Table 2. The results reveal that there are variations in the fiber volume fraction of human PDL across different regions. The volume fraction of collagen fiber in the apex was about 51.988%, the middle was about 63.142%, and the neck was about 60.312% according to the average of five sections. The collagen fiber volume fraction was the highest in the middle region of the PDL, followed by the neck, and the lowest in the apex region.

### 4.2. Uniaxial Tensile Test Results

In practical engineering applications, the first Piola–Kirchhoff stress (P) can usually be measured directly. The relationship between it and the Cauchy stress (σ) is as follows:

(25) $$\sigma =J^{-1}\cdot P\cdot F^{T}$$

Therefore, $\sigma_{11}$ is as follows:

(26) $$\sigma_{11}=\lambda \frac{F_{u}}{A_{0}}$$

$F_{u}$ represents the tensile force applied to the samples in a uniaxial tensile test. $A_{0}$ represents the initial sample area.

**Figure 5:**
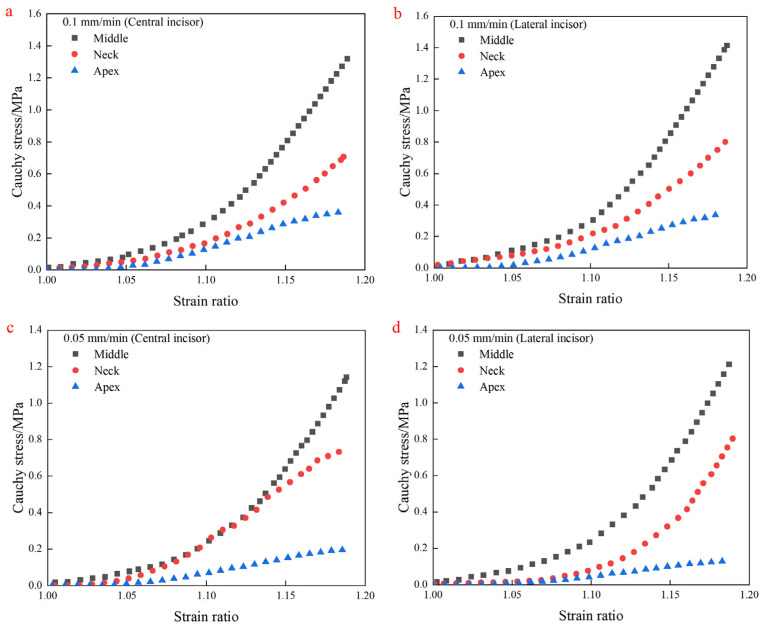

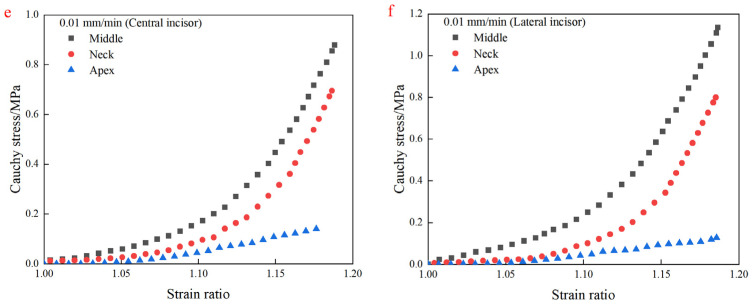
Uniaxial tensile test results at different rates. (**a**,**b**) Test results of central incisor and lateral incisor samples at rate of 0.1 mm/min; (**c**,**d**) test results of central incisor and lateral incisor samples at rate of 0.05 mm/min; (**e**,**f**) test results of central incisor and lateral incisor samples at rate of 0.01 mm/min.

Figure 5 shows the uniaxial tensile test results. The results show that different regions of the human PDL all showed significant non-linear elastic mechanical properties. The Cauchy stress–strain ratio curve tends to be flat in the initial stage of stretching relatively, and the stress increase is not significant when the strain ratio is less than 1.05. This stage can be regarded as a linear relationship, and the stress levels in different regions of the PDL are similar. With the deepening of the test, the slope of the Cauchy stress–strain ratio curve increases significantly when the strain ratio is more than 1.1, and the stress level increases significantly, showing significant hyperelastic characteristics. The difference in stress levels in different regions of PDL gradually increased with the increase in strain ratio. The trend and characteristics of the central incisor and lateral incisor were similar.

### 4.3. Fitting Results

Fit the hyperelastic constitutive model to the experimental data to further validate the accuracy of the model. Based on the analysis of the experimental data, the existing six-parameter model exhibits a significant issue of parameter redundancy. Therefore, this paper simplifies the six-parameter model into a three-parameter non-redundant model. Through multiple fittings, the most reasonable linear constraints have been obtained. The linear constraints are as follows:

(27) $$\left\{\begin{array}{l} c_{2}=0.39571c_{1}+6.30991\\ c_{4}=-5.38351c_{3}-8.07826\\ c_{6}=-5.53492c_{5}+4.62974 \end{array}\right.$$

**Figure 6:**
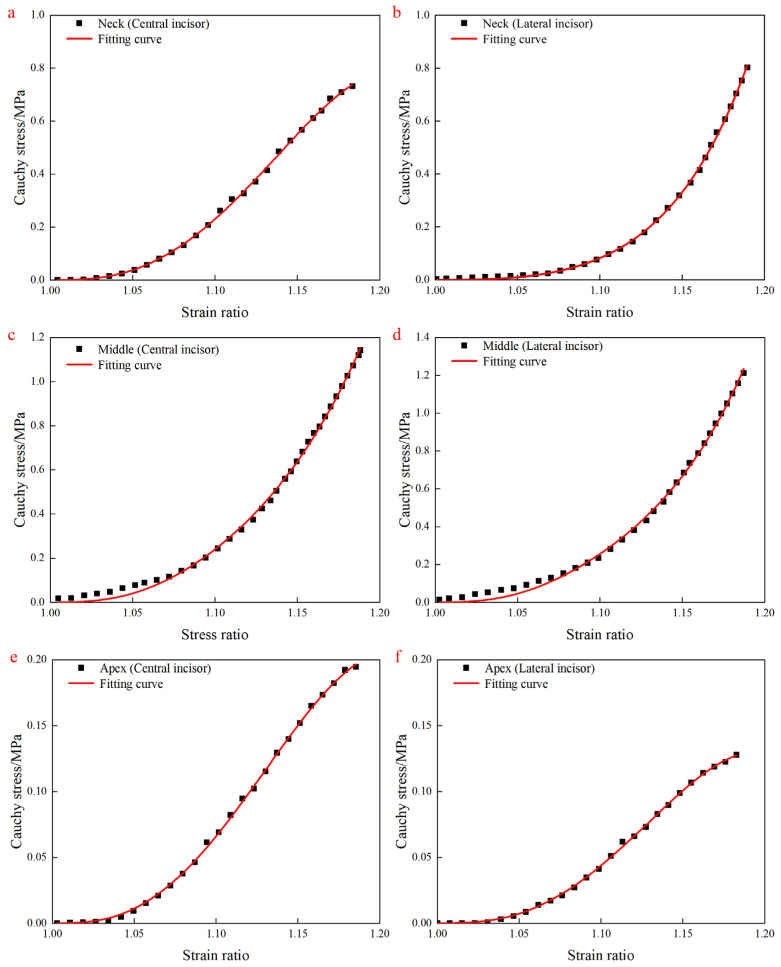
The fitting results. (**a**,**b**) Fitting results of central incisor and lateral incisor samples in the neck region; (**c**,**d**) fitting results of central incisor and lateral incisor samples in the middle region; (**e**,**f**) fitting results of central incisor and lateral incisor samples in the apex region.

The goodness of fit (R^2^) was all greater than 0.99. The fitting results shown in Figure 6 confirmed that the constitutive model could characterize the hyperelastic mechanical properties and could predict the stress changes in PDL well according to the volume fraction of collagen fibers. These parameters of the hyperelastic constitutive model can be obtained by fitting. These parameters are shown in Table 3.

## 5. Discussion

The stress level in the middle region was the highest, and the maximum stress value reached 1.41 MPa. The collagen fiber volume fraction in the middle region was the highest, which was the main reason for the strongest resistance to tensile load according to the measurement results of the volume fraction. The collagen fiber volume fraction of the neck samples was second, and its tensile load resistance was lower than that of the middle region but higher than that of the apex region. The maximum stress level of the neck samples at different rates and tooth positions ranged from 0.71 to 0.80 MPa. The stress level of the apex region was the lowest compared with that of the other parts of the sample. The overall stress level ranged from 0 to 0.36 MPa, which was related to the smallest volume fraction of collagen fibers. The PDL collagen fibers in the apex region are distributed radially. The angle between the collagen fibers’ spatial distribution direction and the loading direction of the uniaxial tensile test was too large. As a result, its load-bearing tensile capacity is the lowest. The collagen fibers in the neck region are arranged horizontally, which can effectively resist unidirectional load. The collagen fibers in the middle region are arranged in a diagonal interwoven distribution, which can effectively enhance their ability to resist load [15,23].

The difference in collagen fiber volume fraction is mainly due to the different functions of PDL in different regions. The PDL in the apex region has the lowest collagen fiber volume fraction. Because it plays a positioning role mainly in the daily use of teeth instead of resisting tensile loads. Healthy teeth have certain physiological mobility during daily chewing behaviors, and the distance between the rotation center of a single root tooth and the alveolar crest is 1/3 [30,31]. The amount of movement in the neck region is greater compared to other regions when the tooth rotates. Therefore, compared to the middle region, the PDL in the neck region should have lower tensile load resistance to allow for enough physiological mobility after stress. Excessive resistance can negatively impact dental health [32]. Consequently, the collagen fiber volume fraction in the neck region is lower than that in the middle region. The middle PDL region primarily absorbs complex forces from daily chewing, resulting in the highest anti-tensile capacity and the highest collagen fiber volume fraction [23].

Different regions of the PDL exhibited similar mechanical properties at various rates across different tooth positions. In the middle region of the central incisor, maximum stress values were 1.32, 1.14, and 0.88 MPa at rates of 0.1, 0.05, and 0.01 mm/min, respectively. In the apex region, the values were 0.36, 0.19, and 0.14 MPa, while in the neck region, they were 0.73, 0.71, and 0.69 MPa. Higher tensile load rates in the middle and apex regions enhanced the characterization of hyperelastic mechanical properties. This indicates that the PDL in the neck region is less influenced by the load movement rate, while the middle and apex regions are more affected by the load movement rate. However, the maximum stress value of the PDL in the middle region at a rate of 0.01 mm/min is still higher than that of the PDL in the neck region at a rate of 0.1 mm/min. This indicates that the load movement rate has a certain influence on the characterization of the hyperelastic mechanical properties of the PDL, but the collagen fiber volume fraction of PDL is still the main factor influencing the hyperelastic mechanical properties of PDL.

The PDL showed some viscosity at lower rates, which was more significant in areas with low collagen fiber volume fraction, such as the apex region [33,34]. Under low-speed loading, the steep slope of the apex region stress ratio-strain curve appears later, and the steep slope height is lower. The different regions of lateral incisor teeth also show similar trends and characteristics. However, the specific performance is still different, given that the different functions of different tooth positions lead to different mechanical properties.

The current research still has some limitations. First, this research focuses on the hyperelastic mechanical properties of the PDL, neglecting its viscoelastic mechanical properties. Future work should consider both the hyperelasticity and viscoelasticity of collagen fibers in order to establish a visco-hyperelastic constitutive model that more accurately describes the mechanical properties of the PDL. Second, in orthodontic treatment, the loading conditions on the PDL are more complex. The PDL is inevitably influenced by shear forces. To more accurately describe the mechanical performance of the PDL under orthodontic forces, it is essential to investigate its shear properties. Integrating shear performance to develop a more precise mechanical model will also be a key focus and direction for future research.

## 6. Conclusions

The results of uniaxial tensile tests indicate that the PDL in different regions exhibits significant hyperelastic mechanical properties under instantaneous tensile loads. The collagen fiber volume fraction is one of the main factors influencing the hyperelastic mechanical properties of the PDL. The higher the collagen fiber volume fraction, the more pronounced the hyperelastic mechanical properties of the PDL. The hyperelastic constitutive model constructed in this research can describe these hyperelastic mechanical properties effectively. This research has certain theoretical value for further investigations into the micro-mechanical properties of the periodontal ligament. It also provides some guidance for predicting and analyzing tooth movement in clinical orthodontic treatments, such as the retraction of maxillary teeth.

## Figures and Tables

**Table 1:** Sample dimensions of alveolar bone–PDL–dentin.

	Neck (Central)	Middle (Central)	Apex (Central)	Neck (Lateral)	Middle (Lateral)	Apex (Lateral)
Length/mm	2.53	2.60	2.23	2.27	2.40	2.57
Width/mm	2.16	2.08	2.20	2.22	1.96	2.03
Thickness/mm	0.25	0.25	0.25	0.25	0.25	0.25
Area/mm^2^	5.4648	5.4080	4.9060	5.0394	4.7040	5.2171

**Table 2:** Volume fraction of collagen fibers in each slice from different regions.

$V_{f}$ (%)	Section 1	Section 2	Section 3	Section 4	Section 5	Average
Neck	60.007	51.991	53.889	67.895	67.78	60.312
Middle	58.249	65.771	65.65	61.369	64.669	63.142
Apex	62.198	59.01	41.2	51.417	46.116	51.988

**Table 3:** Hyperelastic constitutive model parameters.

	$c_{1}$/MPa	$c_{3}$/MPa	$c_{5}$/MPa	*R* ^2^
Neck (central)	25.57	9.76	−6.61	0.998
Neck (lateral)	−7.13	40.93	55.60	0.999
Middle (central)	24.30	−15.23	18.00	0.997
Middle (lateral)	35.33	−16.01	20.90	0.997
Apex (central)	8.12	0.58	1.69	0.999
Apex (lateral)	8.31	7.05	−4.27	0.999

## Data Availability

The original contributions presented in this study are included in the article. Further inquiries can be directed to the corresponding authors.
